# Supplementary figures and images for: Antisense oligonucleotides to therapeutically target SARS-CoV-2 infection
Source: PLoS One. 2023 Feb 3;18(2):e0281281. doi: 10.1371/journal.pone.0281281 (PMC9897518; doi:10.1371/journal.pone.0281281)

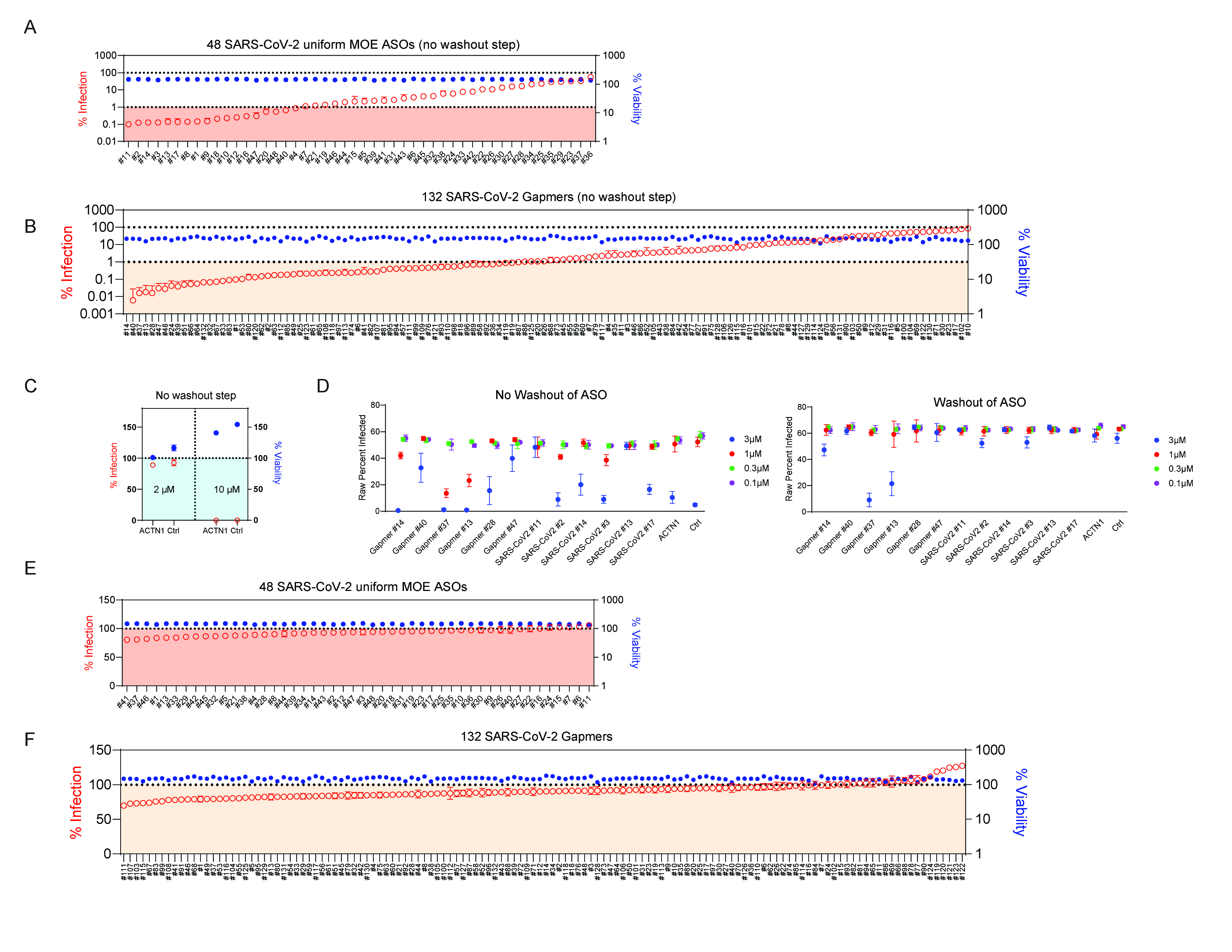

Supplement: S1 Fig — (A) SARS-CoV-2 bioassay screening for 48 uniform MOE ASOs targeting SARS-CoV-2 genome against WA1 strain in H1437 cells without washout step. (B) SARS-CoV-2 bioassay screening for 132 Gapmers targeting SARS-CoV-2 genome against WA1 strain in H1437 cells without washout step. (C) SARS-CoV-2 bioassay for control (Ctrl) ASO and ACTN1 ASO without washout step against WA1 strain in H1437 cells showing non-specific activity at 10 μM. (D) Comparison of selected SARS-CoV-2 ASOs with or without washout step against WA1 strain in H1437 cells. (E) SARS-CoV-2 bioassay screening for 48 uniform MOE ASOs targeting SARS-CoV-2 genome with washout step against WA1 strain in H1437 cells. (F) SARS-CoV-2 bioassay screening for 132 Gapmers targeting SARS-CoV-2 genome with washout step against WA1 strain in H1437 cells. (TIF) [file pone.0281281.s001.tif]
