# Supplementary material for: Antisense oligonucleotides to therapeutically target SARS-CoV-2 infection
Source: PLoS One. 2023 Feb 3;18(2):e0281281. doi: 10.1371/journal.pone.0281281 (PMC9897518; doi:10.1371/journal.pone.0281281)
Supplement: Attachment — Submitted filename: Response to Reviewers.docx [file pone.0281281.s003.docx]

**Response to Reviewers**

**Reviewer #1:** The authors have shown that ASOs -Gapmer and MOE modified ASOs are strong inhibitors of SARS-COV2 infection. The authors need to perform further experiments to validate their fundings.

1. Did the authors check for toxicity of these ASOs?

*>> We have checked the cell viability in parallel with infectivity after every ASO treatment, and the results have been provided in figures with the left-axis indicating infectivity and right-axis indicating viability. No significant impairment of cell viability was observed.*

2. What was the duration and MOI for virus infection?

*>> The treatment regimen is illustrated in Figure 1C. In detail, the duration of virus exposure is 48 hours after 24 hours pretreatment of ASOs. The SARS-CoV-2 infection MOI was described in the Materials and Methods section under the sub-section entitled “SARS-CoV-2 high-content bioassay”. In H1437 cells, MOI=1 for both WA1 and B.1.1.7 variants. In iAEC2 cells, MOI=10 for B.1.1.7 variant. The MOIs were determined as those that achieved 60% of raw infection in the target cell line.*

3. Do authors have in vivo proof of concept for their ASOs?

*>> Antisense technology has been used in several disease models in human clinical trials, such as 2’MOE ASOs in phase III trials of Amyotrophic lateral sclerosis and* *Huntington’s disease, as well as cET Gapmers in phase II trials of cystic fibrosis and prostate cancer (S.T. Crooke, et al.*, *Journal of Biological Chemistry, 2021). The dosing route of ASOs is ready to be formulated for inhalation. That said, we do not have in vivo proof of concept in this particular manuscript as it was beyond the scope of the study.*

4. Abstract is very incoherent. It’s been a few years nor several years of pandemic.

*>> We have revised the abstract to address this.*

5. Authors should also check for virus titers after treating with ASOs.

*>> SARS-CoV-2 virus was propagated using VeroE6 cells, and viral titer was determined prior to the SARS-CoV-2 bioassay. The in vitro SARS-CoV-2 bioassay is to transduce cells without the presence of polybrene or any other polycation. Thus, the culture supernatant is saturated with virus to achieve around 60% of raw infection without causing cytopathogenic effects to the target cells. MOIs were optimized for each cell line and various SARS-CoV-2 variants. We identified the infected cells by immunofluorescence staining of nucleocapsid protein of SARS-CoV-2 virus with or without various ASO treatments, and the free virus in the cell culture supernatant was not a good indicator of infectivity in the SARS-CoV-2 bioassay.*

**Reviewer #2:** The manuscript titled ‘Antisense oligonucleotides to therapeutically target SARS-CoV-2 infection’ is well written in English. The authors screen ACE2 and TMPRSS2 ASOs to inhibit the infection of SARS-CoV-2 variants. The study demonstrates the usefulness of the approach, and it can be used in other disease models as it is a very specific response unlike certain drugs.

*>> We thank the reviewer for their positive comments. We agree that ASOs are an exquisitely specific targeting approach with broad applications in other diseases.*

The authors have not performed any statistical analysis which is visible on the graphs for reduction of viral infection using the ASOs, which is a major flaw to understand if the data is significant.

*>> We thank the reviewer for this important point. In the revised version, we have calculated p values and provided the exact p values on figures for better understanding of the significance.*
